# Surgical management of multiple congenital epulis in the maxillary and mandibular alveolar ridges of a newborn: A case report

**DOI:** 10.1016/j.ijscr.2024.110579

**Published:** 2024-11-15

**Authors:** Khadijeh Sadat Najib, Hamide Barzegar, Mehrdad Rezaei, Mahsa Kohandel-Shirazi, Marzieh Davoodi

**Affiliations:** a Neonatal Research Center, Shiraz University of Medical Sciences, Shiraz, Iran; b Assistant Professor of Pathology, Department of Pathology, Shiraz University of Medical Sciences, Shiraz, Iran; c Neonatal Research Center, School of Medicine, Shiraz University of Medical Sciences, Shiraz, Iran

**Keywords:** Epulis, Newborn, Surgery case report

## Abstract

**Introduction and importance:**

Congenital epulis is a rare and benign newborn tumor. There are some papers on this entity; however, very few reports focus on its macroscopic view.

**Case presentation:**

We present a newborn girl with multiple congenital epulis of the mandibular and maxillary alveolar ridges who underwent a successful surgical intervention in a resource-limited setting.

**Clinical discussion:**

Congenital epulis is frequently diagnosed by histopathological examination, although sonography could be helpful before birth. Despite the benign nature of the disease, immediate intervention is often required as it can prevent feeding and cause asphyxia in neonates. Surgical excision is the standard treatment.

**Conclusions:**

Congenital epulis can be identified clinically shortly after birth. They cause a substantial surgical challenge due to multiple or large lesions and problems with breathing and feeding. Surgical repair must be performed as early as possible.

## 1 Introduction

Congenital epulis is a rare and benign lesion of the gingiva [1], also referred to as granular cell tumor of the gingiva, congenital granular cell tumor, congenital granular cell fibroblastoma, congenital granular cell myoblastoma, Neumann's tumor, and congenital granular epulis [2]. Although the exact pathogenesis of the lesion remains elusive, it is hypothesized to originate from epithelial cells, undifferentiated mesenchymal cells, pericytes, fibroblasts, smooth muscle cells, or nervous system cells [3]. The condition is seen in roughly 0.0006 % of births [4], typically along the maxillary alveolar ridge in white neonate girls. Congenital malformations or teeth deformities are absent, except for one reported case of a co-occurring genital anomaly. Although most cases are solitary, problems with breathing and feeding may emerge if multiple or large lesions are present [5]. Despite the benign nature of the disease, immediate intervention is often required as it can prevent feeding and cause asphyxia in neonates [4]. Surgical excision is the standard treatment, though tiny lesions may regress spontaneously [6].

## 2 Case presentation

**Fig. 1:**
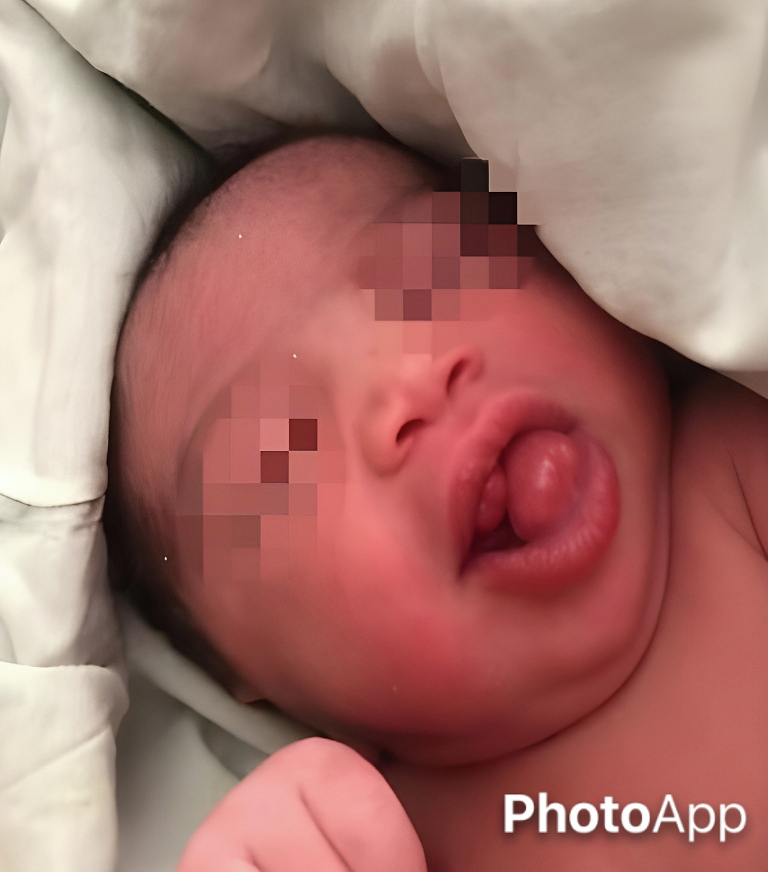
Multiple masses are present in the maxillary and mandibular alveolar ridges.

A term female neonate was brought to our center immediately after delivery due to the presence of two firm, pedunculated masses protruding from her mouth [Fig. 1]. She was born via Cesarean section, weighing 3200 g, with a height of 51 cm, head circumference of 36 cm, and Apgar scores of 8 and 9 at 1 and 5 min, respectively. The mother was a 36-year-old woman with a history of two successful pregnancies and no abortions. She also had a history of gestational diabetes. The parents were relatives, but there was no family history of hereditary diseases. Prenatal ultrasound revealed a lesion measuring 30 × 19 mm in the anterior part of the fetal mouth, which kept the fetal mouth open and led to polyhydramnios.

On examination, two smooth, pedunculated masses measuring approximately 2.0 × 2.0 cm were attached to the maxillary and mandibular alveolar ridges, sharing the same color as the oral mucosa. These masses were firm, non-compressible, non-reducible, and non-tender on palpation. The neonate was unable to close her mouth. Due to the obstruction caused by the lesions, breastfeeding was impossible, and a nasogastric tube was inserted for feeding. There were no signs of airway obstruction, respiratory distress, or other structural deformities, and mechanical respiratory support was not required.

**Fig. 2:**
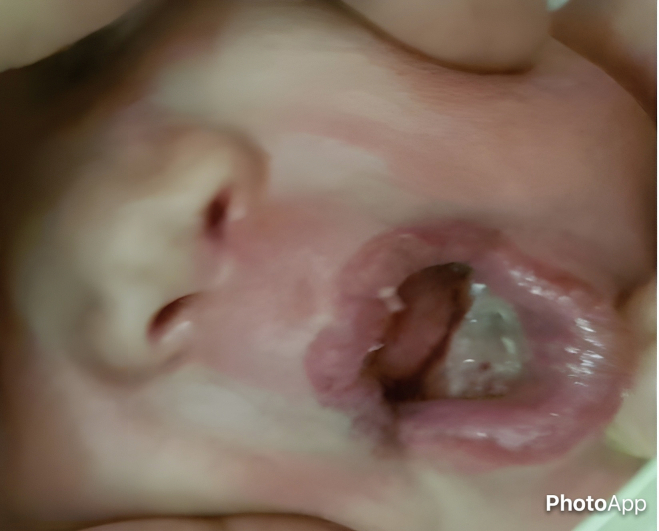
Postoperative clinical image demonstrating the site of the wound.

Five days after birth, the patient underwent surgical excision of the masses under general anesthesia with oral intubation due to the large size and pedunculated nature of the tumors. The masses were attached to the alveolar mucosa. The procedure was uneventful, with no significant bleeding during or after surgery. Oral breastfeeding was initiated on the first postoperative day, and the child was discharged the following day [Fig. 2].

**Fig. 3:**
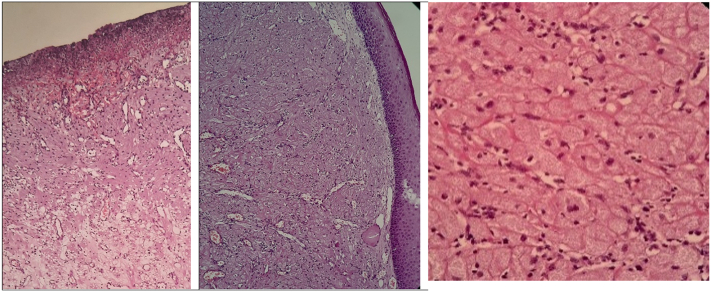
Microscopic study showed subepithelial sheet of large polygonal cells with granular eosinophilic cytoplasm and small round nuclei with inconspicuous nucleoli. There is a prominent delicate vasculature and focal ulceration of the surface epithelium. Psuedoepitheliomatous hyperplasia is absent.

Histological examination of the specimens revealed prominent delicate vasculature and focal ulceration of the surface epithelium, without evidence of pseudoepitheliomatous hyperplasia. Microscopic analysis showed subepithelial sheets of large polygonal cells with granular round nuclei and inconspicuous nucleoli, consistent with congenital epulis [Fig. 3].

At a follow-up visit one week later, the infant had no aesthetic or feeding problems. By 12 months of age, she had normal dentition with four teeth, and the parents had no complaints.

The work has been reported in line with the SCARE criteria.

## 3 Discussion

Congenital epulis of the newborn is a rarely encountered gingival tumor, first described by Neumann in 1871 and inheriting its name from a Greek term meaning “on the gum” [2]. A single lesion is typically seen on the alveolar ridge, though multiple lesions are present in 5–16 % of cases. In cases of multiple lesions, the maxilla or mandible is affected, with airway obstruction possible. Roughly a tenth of cases show simultaneous involvement of the maxillary and mandibular alveolar ridges [7]. In our case, multiple masses were present.

The exact incidence of congenital epulis is unknown. One American Center noted only a couple of cases over 21 years, while a tertiary referral center in Wales reported only one case over 28 years [7]. This condition is more prevalent in females, with a ratio of 8:1, and is thought to involve a hormonal mechanism related to estrogen and progesterone receptors [8]. The condition typically does not present with other simultaneous teeth or congenital anomalies [9]. This was also the case with our patient.

Congenital epulis is usually solitary, with multiple lesions reported in about 10 % of cases. In Table 1, we have summarized some of the reported cases of multiple congenital epulis since 2010.

**Table 1:** Review cases of multiple congenital Epulis.

Case	Year published	Demographic	Symptoms	Number and location	Treatment
1 [10]	2011	Female	–	2-maxillary and mandibular alveolar ridge	Surgery
2 [5]	2012	Female Term	Preserved normal mouth closure, feeding difficulty	3- anterior gingiva, mandible	Surgery
3 [11]	2013	Female Term	Feeding difficulty	2- left maxilla	Surgery for the large and self-regression for the small one
4 [12]	2014	Female Term	Feeding difficulty	3- left maxilla and left mandible	Surgery
5 [13]	2015	Female	Feeding difficulty	2- anterior alveolar region	Surgery
6 [14]	2019	Female	Feeding difficulty	2-superior and lower alveolar arch	Surgery
7 [15]	2021	Female Term	Feeding difficulty	3- left maxillary arch and midline mandibular arch	Surgery
8 [16]	2022	Female Term	Feeding difficulty	2- right mandibular arch and posterolingual	Surgery for the large and self-regression for the small one

Congenital epulis may be associated with certain prenatal conditions like polyhydramnios. The lesion(s) may impede feeding, breathing, and the ability to close the mouth [6]. Prenatal polyhydramnios was noted in our case, as was interference with feeding and mouth closure. However, breathing was unaffected.

Congenital epulis usually forms after 22 weeks of gestation [17], at which time it can be diagnosed via 3D ultrasonography or magnetic resonance imaging (MRI) [18]. While the earliest report of an antenatal diagnosis was at 31 weeks of gestation [1], our case's abnormality was also detected at that week.

The differential diagnoses for congenital oral mucosa lesions include teratoma (epignathus), hemangioma, fibroma, choristoma, hamartoma, melanotic neuroectodermal tumor of infancy, rhabdomyoma, rhabdomyosarcoma, lymphangioma, osteogenic and chondrogenic sarcomas, and granular cell tumor [18]. The site of involvement, size, growth velocity, and accompanying lesions are important clinical parameters, with the histopathological examination usually confirming the diagnosis.

The cornerstone of congenital epulis management is surgical excision, performed with local or general anesthesia [6]. Nonetheless, small solitary lesions (<2 cm) may regress spontaneously, as noted in nearly 5 % of the reported cases [1,18]. The treatment selected for our case was surgical excision under general anesthesia to alleviate the newborn's problems with feeding and mouth closure. We chose general anesthesia for our case due to its safety, effective airway protection, and ability to alleviate the stress and pain associated with the surgery. While local anesthesia could reduce complications related to general anesthesia, it may pose challenges in managing a moving and crying child, potentially complicating tumor excision and achieving adequate hemostasis [19,20].

## 4 Conclusion

We should consider epulis tumors in the differential of gingival tumors. As it may affect feeding or respiration, prompt treatment is necessary.

## Consent

Written informed consent was obtained from the patient's parents/legal guardian for publication and any accompanying images. A copy of the written consent is available for review by the Editor-in-Chief of this journal on request.

## Ethical approval

This study was approved by the ethical committee of Shiraz University of Medical Sciences IR.SUMS.MED.REC.1403.284.

## Guarantor

Corresponding author.

## Research registration number

1. Name of the registry: None.
2. Unique identifying number or registration ID: None.
3. Hyperlink to your specific registration (must be publicly accessible and will be checked): None.

## Funding

N/A.

## Author contribution

Hamide Barzegar: Conceptualization, Data Curation, Writing - Original Draft, Writing - Review & Editing. Khadijeh Sadat Najib: Conceptualization, Investigation, Supervision, Writing - Original Draft, Writing - Review & Editing. Mehrdad Rezaei: Conceptualization, Data Curation, Writing - Original Draft, Writing - Review & Editing, Supervision. Mahsa Kohandel-Shirazi : Data Curation, Writing - Original Draft, , Supervision . Marzieh davoodi: Conceptualization, Data Curation, Writing - Original Draft, Writing - Review & Editing, Supervision, Project administration

## Conflict of interest statement

The authors declare that they have no conflict of interest.
